# The value of fetal magnetic resonance imaging in diagnosis of congenital anomalies of the fetal body: a systematic review and meta-analysis

**DOI:** 10.1186/s12880-024-01286-5

**Published:** 2024-05-16

**Authors:** Louise Wilson, Elspeth H. Whitby

**Affiliations:** 1 School of Medicine and Population Health, University of Sheffield, Level 4, Jessop Wing, Tree Root Walk, Sheffield, S10 2SF UK; 2 Medical Imaging and Medical Physics, Sheffield Teaching Hospitals, Sheffield, UK

**Keywords:** Ultrasound, Magnetic resonance imaging, Prenatal diagnosis, Fetal, Congenital anomaly

## Abstract

**Objectives:**

To undertake a systematic review to assess the accuracy of fetal MRI in diagnosis of non-CNS congenital anomalies of the fetal body in comparison with antenatal ultrasound when correlated to postnatal diagnosis.

**Methods:**

Searches were conducted from electronic databases, key journals and reference lists for eligible papers. Inclusion criteria was original research studies comparing the diagnostic results of antenatal ultrasound, fetal MRI and final postnatal diagnosis via imaging, surgery or post-mortem testing. Studies of CNS anomalies were excluded. Studies were assessed for risk of bias by two reviewers working independently and data was then extracted by a single reviewer.

**Results:**

12 studies were included with a total of 361 eligible patients who underwent USS and MRI and had a postnatal diagnosis. USS alone had a diagnostic accuracy of 60.6% whereas MRI had an improved diagnostic accuracy of 86.4%. The overall odds ratio was 0.86 (CI 0.202–1.519 and *p*-value < 0.01).

**Conclusion:**

Fetal MRI makes a significant contribution to accurate diagnosis of congenital abnormalities of the fetal body; especially in genito-urinary anomalies. More research is needed to improve the evidence base for the role of fetal MRI in diagnosis of congenital anomalies in other body systems.

**Supplementary Information:**

The online version contains supplementary material available at 10.1186/s12880-024-01286-5.

## Key points

Antenatal ultrasound is the gold standard for detection of anomalies of the fetal body during pregnancy.

Fetal MRI used in addition to ultrasound is more accurate at detecting anomalies of the fetal body.

Fetal MRI is most useful in detection of bilateral renal disease and assessment of its severity, in cases where oligohydramnios affects ultrasound accuracy and in exclusion of cloacal abnormalities.

This study may be limited by the sample size of included studies.

**Supplementary Information:**

The online version contains supplementary material available at 10.1186/s12880-024-01286-5.

## Introduction

Congenital anomalies not affecting the central nervous system (CNS) occur in approximately 206 per 10,000 UK births [1]. While ultrasound scanning (USS) is recognised as the gold standard for diagnosis of congenital anomalies there is increasing evidence for magnetic resonance imaging (MRI) [2]. Fetal MRI is safe in pregnancy and overcomes some limitations of ultrasound such as poor visualisation of the fetus where there is high maternal body mass index (BMI), in oligohydramnios or atypical fetal position [2].

Significant research has been undertaken concerning the diagnostic accuracy of fetal MRI in anomalies of the fetal brain [3, 4]. There has also been extensive research concerning fetal MRI in prognostication of anomalies such as congenital diaphragmatic hernia [5]. However, systematic review evidence for the role of MRI in diagnosis of abnormalities of the fetal body is lacking and there is no consensus on its role in antenatal counselling and decision making.

## Methods

The aim of this study was to assess whether fetal MRI diagnoses congenital body anomalies more accurately than ultrasound alone and to determine how frequently fetal MRI gives additional information which affects management.

The protocol was developed using guidelines from the Preferred Reporting Items for Systematic Reviews and Meta-Analyses (PRISMA) [6]. It has been registered with the International Prospective Register of Systematic Reviews (PROSPERO no. CRD42022379721).

### Eligibility criteria

The eligibility criteria consisted of primary research of congenital anomalies of the fetal body comparing antenatal ultrasound and fetal MRI findings with postnatal diagnosis. A key requirement was for studies to comment on the diagnostic accuracy of both the fetal ultrasound and MRI separately in comparison with the postnatal findings made by imaging, surgery or post-mortem examination.

Studies of the CNS and studies of imaging in prognostication were excluded. Studies published prior to 2000 were excluded because few centres were using fetal MRI clinically at this time and data prior to this time may have been biased by technological limitations. Case reports and narrative reviews were excluded. Any study with three or fewer patients was excluded as these were considered as case reports. Studies involving research of cardiac fetal MRI were also excluded as this was considered to be a separate entity [7], as cardiac fetal MRI is predominantly performed as research and very few centres offer cardiac fetal MRI as a clinical service.

Studies not reported in English and where translation was unavailable were also excluded. For studies where only abstracts were available the authors were contacted directly to request the full paper; studies were excluded where the full paper was not available.

### Search strategy

A search of electronic databases was undertaken using the search strategy illustrated in appendix 1. Databases searched were Medline (via Ovid 1966-present), Embase (via Ovid 1980-present) and Web of Science (1900-present) [8]. Relevant journals were also searched and references from key papers were examined. The searches were conducted in December 2022.

**Fig. 1:**
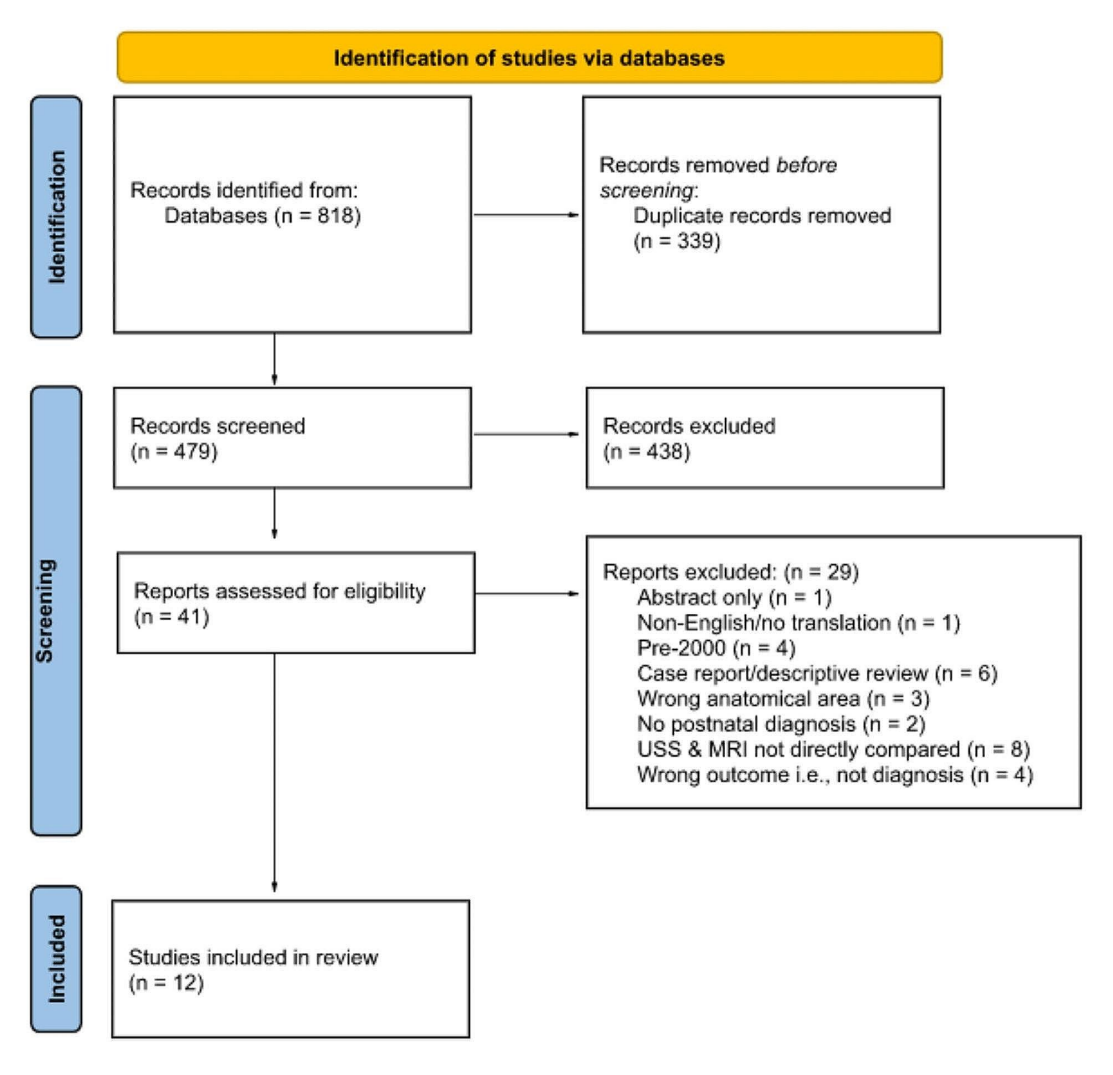
PRISMA flow chart of study selection

Studies were assessed for inclusion by two reviewers working independently and any disagreements were resolved by consensus. A PRISMA flow chart was completed detailing the selection process (Fig. 1).

### Risk of bias assessment

Included studies were assessed for methodological quality using a risk of bias assessment (QUADAS 2 tool) [9]. Risk of bias was assessed by two reviewers working independently and any disagreements were resolved by discussion and consensus. Risk of bias was assessed in the four domains: patient selection, index test, reference standard and flow and timing. Applicability was assessed in terms of patient selection, index test and reference standard. The bias of the index test was considered high in studies where MRI was performed following an inconclusive ultrasound diagnosis. Index test applicability was considered low risk of bias provided ultrasounds performed in other centres were repeated at the tertiary centre prior to MRI and the tertiary ultrasounds were used for the analysis. This was in order to minimise bias caused by variation in sonographer expertise. The use of clinical assessment in reporting of outcome as a reference standard was considered low risk in assessment of bias. This was because although some variability will be introduced this was felt to reflect clinical practice. A time lapse of greater than two weeks between ultrasound and MRI was considered to introduce a high risk of bias; in studies where timings were not specified the risk of bias was deemed unclear.

### Data extraction

Data from the studies was extracted by a single reviewer using a pre-specified data collection tool (appendix 2). Data collected included key study characteristics, the individual diagnostic accuracy of antenatal ultrasound and fetal MRI in comparison with the postnatal diagnosis and how frequently there was agreement or disagreement between the two modalities. For studies which assessed both CNS and non-CNS abnormalities data collection focused on the body anomalies only.

Sensitivity and specificity of the imaging techniques could not be assessed as included studies involved patients referred for fetal MRI scan following an abnormality detected on ultrasound, meaning there were no control groups. The relative odds ratio for the paired MRI and USS diagnostic accuracies were calculated using McNemar’s odds ratio with a 0.5 correction for zero cells. The odds ratios were combined using a random effects model. A funnel plot for assessment of publication bias was also undertaken.

## Results

The searches retrieved 818 studies which were reduced to 479 studies once duplicates were removed. Abstract screening reduced the number of studies to 41 studies which were assessed for eligibility. Following assessment, twelve studies were included in the final analysis. Details of the reasons for exclusion can be found in Fig. 1. PRISMA flow chart. All included studies compared the diagnosis made on ultrasound with a fetal MRI which was performed after the ultrasound anomaly had been detected. This process reflects clinical practice and allowed assessment of whether the MRI provided additional information which altered the management of the pregnancy.

### Study characteristics

The twelve included studies and their characteristics are listed in Table 1. The studies were published between 2003 and 2021, with the majority (*n* = 9) being published after 2009. Three studies [10, 11] were prospective and the remainder [12–19] were retrospective. Two studies [11, 13] specified consecutive patient recruitment whereas the remaining studies [10, 20, 12, 14–19] did not specify the recruitment process.

**Table 1:** Included studies and their characteristics

Author & Year	Title	Country of study	Population	Method of selection	Retrospective (R) or Prospective(P)	No. patients in study	No. patients included in review
Abdelazim 2010	Complementary roles of prenatal sonography and magnetic resonance imaging in diagnosis of fetal renal anomalies	Egypt	Renal anomalies	Unclear	P	20	18
Alamo 2010	Fetal MRI as complement to US in the diagnosis and characterization of anomalies of the genito-urinary tract	Switzerland	Genito-Urinary tract anomalies	Unclear	R	15	15
Alamo 2013	Comparison of foetal US and MRI in the characterisation of congenital lung anomalies	Switzerland	Congenital lung malformation	Consecutive	R	30	26
Barseghyan 2008	Complementary Roles of Sonography and MRI in assessment of fetal urinary tract anomalies	USA	Renal anomalies	Unclear	R	39	39
Behairy 2015	Diagnostic value of fetal MRI in evaluating fetal urinary anomalies	Egypt	Urinary tract anomalies	Unclear	P	30	30
Breysem 2003	The value of fast MR imaging as an adjunct to ultrasound in prenatal diagnosis	Belgium	Brain, neck/chest and abdominal anomalies	Unclear	R	40	14
Crivelli 2021	Contribution of magnetic resonance imaging to the prenatal diagnosis of common congenital vascular anomalies	Switzerland & France	Vascular malformations	Unclear	R	24	24
Gupta 2010	The role of magnetic resonance imaging in fetal renal anomalies	India	Renal anomalies	Consecutive	P	86	27
Hugele 2015	Does prenatal MRI enhance fetal diagnosis of intra-abdominal cysts?	France	Abdominal cysts	Unclear	R	56	49
Ji 2018	Magnetic resonance imaging for evaluation of foetal multicystic dysplastic kidney	China	Multicystic dysplastic kidneys	Unclear	R	55	53
Kajbafzadeh 2007	Comparison of magnetic resonance urography with ultrasound studies in detection of fetal urogenital anomalies	Iran	Genito-Urinary tract anomalies	Unclear	R	342	46
Millischer 2017	Fetal MRI compared with ultrasound for the diagnosis of obstructive genital malformations	France	Genital malformations	Unclear	R	20	20

Seven studies [10–12, 14, 16, 17] investigated renal or urinary tract anomalies. Two studies [13, 15] looked at anomalies of the fetal chest, another two studies [15, 21] focused on abdominal anomalies, one study [18] examined vascular anomalies and one [19] was investigating fetal genital anomalies. None of the included studies involved cervical masses, although these were not specifically excluded.

The median gestation at the time of ultrasound was 28.5 weeks as given in two studies [19, 20]. The gestational age at the time of fetal MRI was stated in four studies [12, 13, 19, 20] which had a combined median gestation of 29 weeks.

The twelve included studies looked at a total of 757 patients. 361 patients (47.7%) were included in this review as 300 did not undergo fetal MRI, five were lost to follow-up, 82 had CNS anomalies and nine had no post-natal diagnosis for comparison. Of the 300 patients who did not undergo fetal MRI 296 came from one study of urinary tract anomalies [17] in which there was a total of 342 patients but only 46 were referred for fetal MRI. The other four patients who did not undergo fetal MRI were in a study of lung malformations [13]. The reasons for including these patients in the studies and not referring these patients for MRI was not clear.

### Methodological quality

**Fig. 2:**
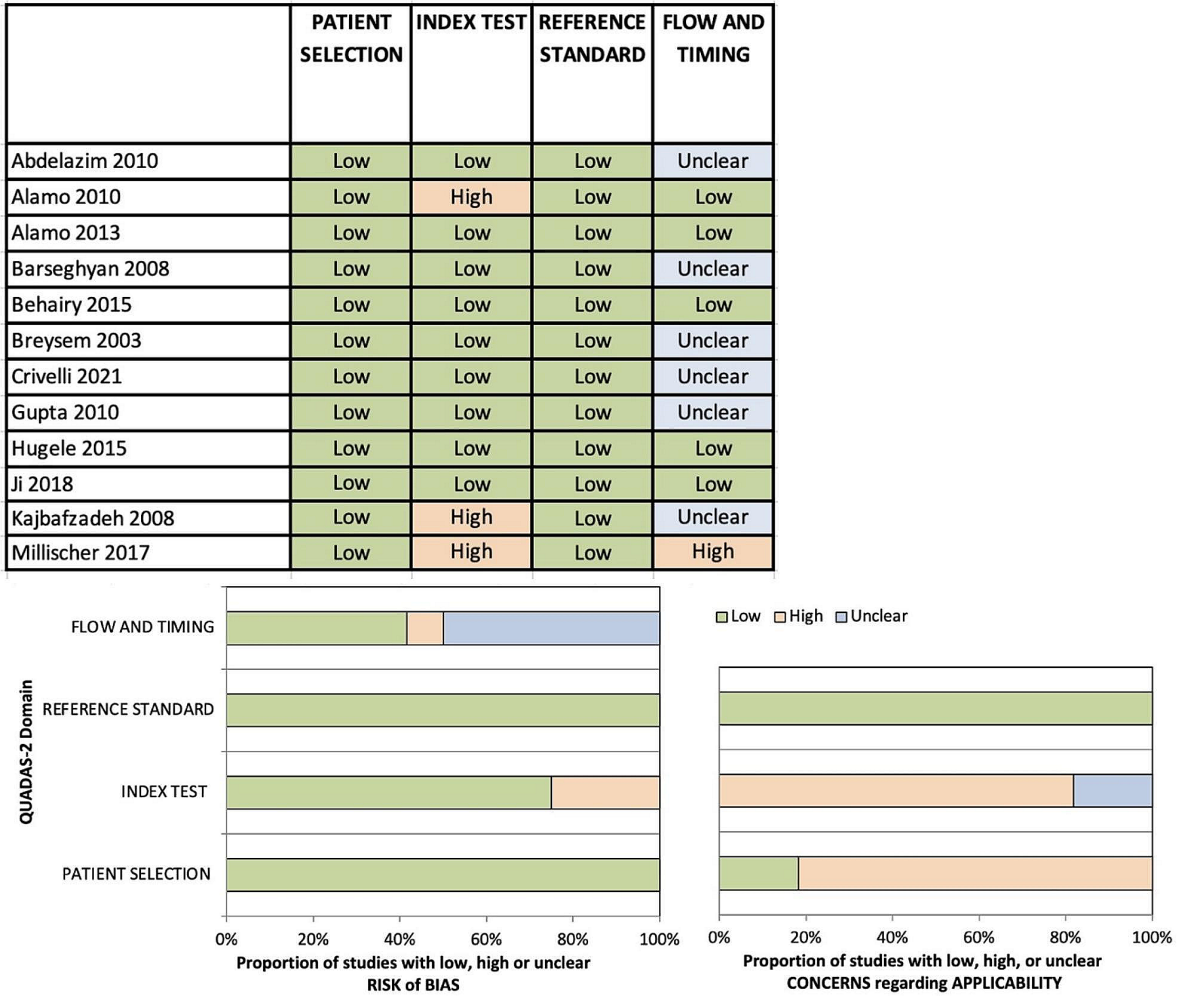
Risk of bias and assessment of applicability using Quadas-2 tool

The methodological quality of included studies was assessed using the Quadas-2 tool [9] and results are summarised in Fig. 2. The risk of bias in patient selection was considered low risk in all studies as studies with unsuitable patients had been excluded.

Risk of bias concerning the index test was high in 3/12 studies [12, 17, 19] where MRI scans were performed due to inconclusive ultrasound results and was low risk in the remaining nine studies [10, 11, 13–16, 18, 21]. Risk of bias introduced by the reference standard was low risk in all studies as ultrasounds were repeated by the tertiary centres performing the MRIs and the diagnoses made from these ultrasounds were used in the analysis. The risk of bias relating to flow and timing was determined by the time between ultrasound and MRI scan; this was low risk in 5/12 [20, 12–14, 21], unclear in 6/12 [10, 11, 15–18] where scan timings were not given and high risk in 1/12 [19] where there was more than two weeks between ultrasound and MRI scan.

### Diagnostic accuracy of USS and MRI

**Fig. 3:**
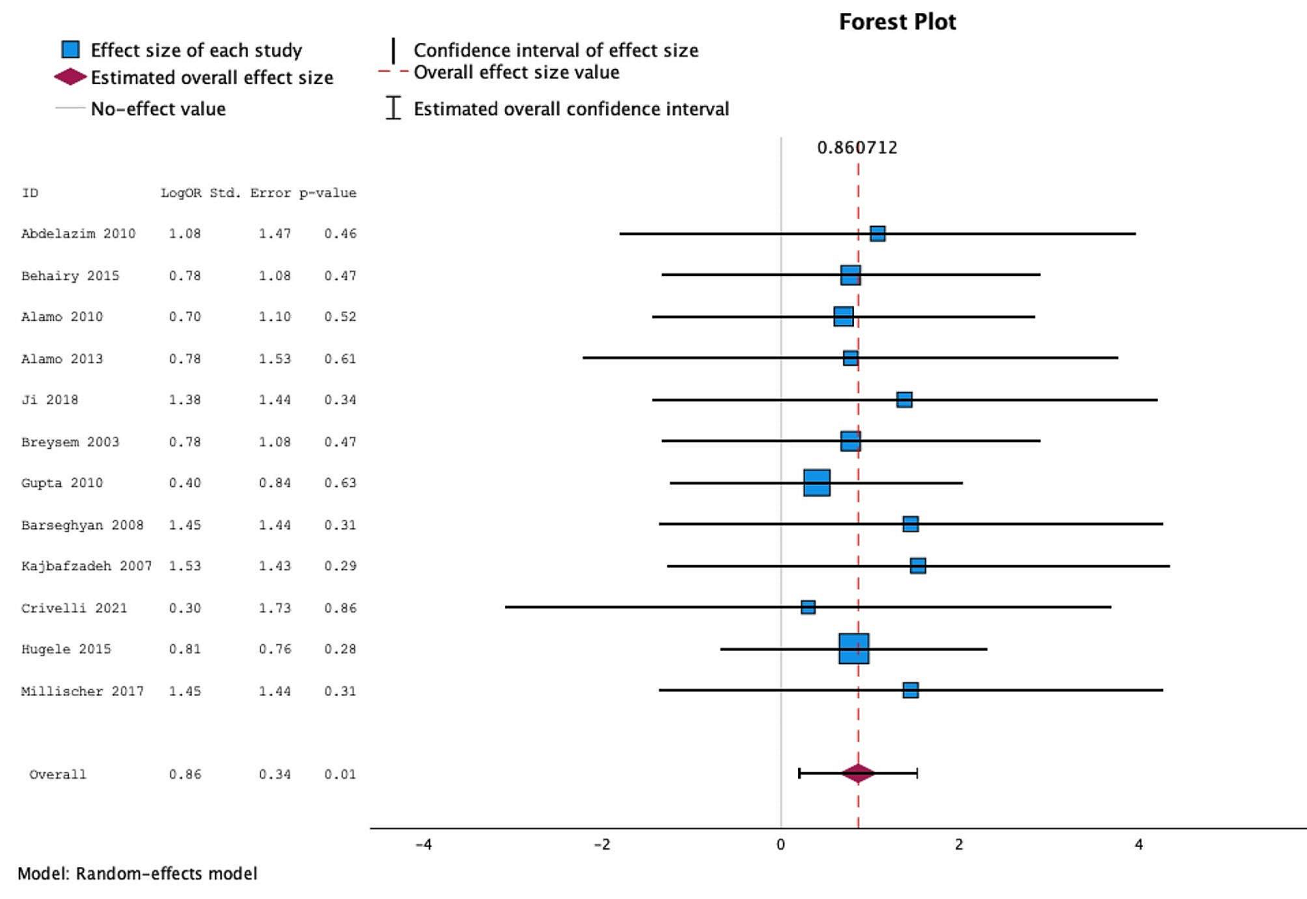
Forest plot of odds ratios for individual studies

The diagnostic accuracy across all twelve studies combined when imaging diagnosis was compared with postnatal diagnosis was 60.6% (219/361) for antenatal ultrasound and 86.4% (312/361) for fetal MRI. All studies showed an improvement in diagnostic accuracy following fetal MRI scan and despite heterogeneity the overall odds ratio when studies were combined was 0.86 (95% confidence interval 0.202–1.519 and *p*-value < 0.01). The forest plot of the relative odds ratios for each study is shown in Fig. 3.

**Fig. 4:**
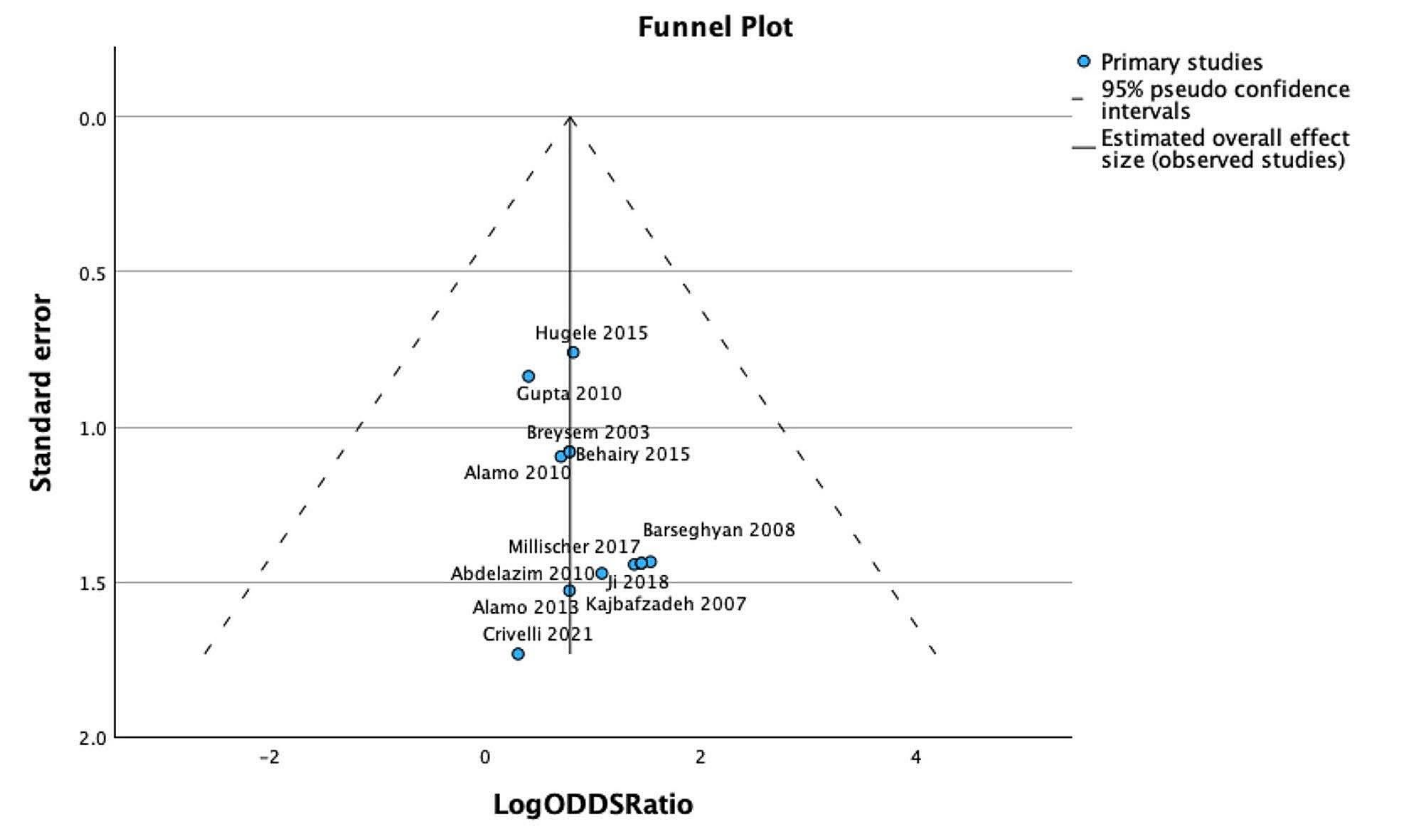
Funnel plot of log Odds ratio and standard error for individual studies

A funnel plot was generated for assessment of publication bias which showed reasonable symmetry meaning it is less likely any bias or heterogeneity within the meta-analysis is significantly affecting the results. This is detailed in Fig. 4.

The seven studies investigating renal and urinary tract abnormalities [10–12, 14, 16, 17] reported a combined accuracy of 68% (155/228) for ultrasound and 94% (214/228) for MRI. The two studies of chest anomalies [13, 15] found the diagnostic accuracy to be 40% (12/30) and 53% (16/30) for ultrasound and MRI respectively. The abdominal studies [15, 21] reported the diagnostic accuracy as 49% (29/59) for ultrasound and 76% (45/59) for MRI. The study looking at detection of vascular anomalies [18] found similar results between ultrasound and MRI in terms of diagnostic accuracy; the ultrasound diagnosis was correct in 71% (17/24) and MRI was correct in 75% (18/24). When this study split their results into detection of lymphatic malformations and haemangiomas separately, they concluded the same rates of diagnostic accuracy for haemangiomas, which were poorly described by both imaging modalities, as 25% (1/4) and a marginally improved rate with MRI diagnosis of lymphatic malformations (ultrasound 16/20 correct and MRI 17/20 correct). The study of obstructive genital malformations [19] had a relatively small sample size of 20 patients but showed a significant difference in diagnostic accuracy between ultrasound (30% or 6/20) and MRI (95% or 19/20). This was predominantly due to the ability of MRI to correctly exclude cloacal abnormalities.

### Agreement between USS and MRI

Antenatal ultrasound and fetal MRI were in agreement with each other and the final postnatal diagnosis in 59% (213/361) of cases. In 6.4% (23/361) the ultrasound and MRI were in agreement but gave an incorrect diagnosis compared with the final outcome. This discordance was most pronounced in the studies assessing chest lesions [13, 15] where ultrasound and MRI agreed but were wrong in 40% of cases (12/30). This was primarily complex lung lesions where both imaging modalities gave non-specific findings.

### Change in diagnosis following MRI

The MRI diagnosis correctly changed the ultrasound diagnosis i.e. the MRI was in concordance with the postnatal outcome diagnosis but ultrasound was incorrect in 28% of cases (101/361). This was most notable in the abdominal studies [15, 21] in which MRI correctly changed the diagnosis in 30.5% (18/59) and in the renal/urinary tract studies [10–12, 14, 16, 17] in which 28% (64/228) of the ultrasound diagnoses were correctly changed by MRI.

In 1.7% of fetuses (6/361) the MRI scan incorrectly changed the diagnosis given by the ultrasound. This was again noted in the abdominal studies [15, 21] and urinary tract studies [8–20, 12, 14, 15] in which the MRI gave an incorrect diagnosis but the initial ultrasound report was in agreement with the postnatal diagnosis.

### Additional information provided by MRI and change in management

**Fig. 5:**
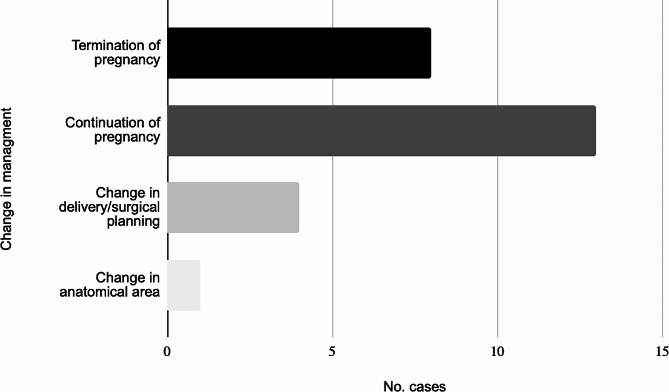
Cases with change in management following fetal MRI scan (*n* = 26)

The MRI scans gave additional diagnostic information in 26.8% of fetuses (93/347) as reported by eleven of the twelve studies; this information was not clearly given in one study [15]. Seven studies [10–13, 16, 19] commented on the number of cases where the additional information provided by the fetal MRI changed the management of the pregnancy. They found antenatal management was influenced by the MRI report in 14.9% of cases (26/175) as illustrated in Fig. 5. This was most significant in the study of obstructive genital malformations [19] in which management was changed in 14/20 cases (70%). The change in management consisted of termination of pregnancy (*n* = 8), continuation of pregnancy (*n* = 13), plans for immediate delivery and postnatal management/surgery (*n* = 4) and a change in body system anomaly diagnosed (*n* = 1).

All studies except two [15, 16] commented on the anomaly or diagnosis in which the addition of MRI was felt to have the most benefit. MRI was concluded to be the most useful in detection and severity of bilateral renal disease in three studies [10, 14, 20], in detection of fetal pelvic anomalies [12], in cases where oligohydramnios affected ultrasound scan accuracy [11] and in exclusion of cloacal anomalies [19].

## Discussion

This review has demonstrated an increase in diagnostic accuracy of 25.8% in congenital anomalies of the fetal body with the use of fetal MRI compared with antenatal ultrasound alone in relation to the final postnatal diagnosis. Despite focusing on congenital anomalies of different areas of the fetal body each study reported an overall increase in diagnostic accuracy with fetal MRI and the combined odds ratio was 0.86 (CI 0.202–1.519 and *p* value < 0.01). Additional information was provided by the MRI in 26.8% and management was changed in 14.9%. There were a small number of cases in which the MRI incorrectly changed the diagnosis and was discordant with the postnatal diagnosis (1.7%).

This data highlights the importance of the use of fetal MRI as an adjunct to clinical expertise and the views of families when making decisions regarding the management of a pregnancy. This is shown most prominently in the seven studies which reported an overall change in management in 14.9% of cases based on the results of the MRI. These changes in management led to continuation of pregnancy in 50% of the 26 cases discussed and 30% of families opting for termination of pregnancy following the change in diagnosis. The additional information provided allowed precise planning of delivery and postnatal management in 15%.

Previous research has shown that image quality in fetal MRI is less affected by high maternal BMI, atypical fetal position and oligohydramnios than in antenatal ultrasound [2]. While it is clear there is some impact by these factors, there is a role for fetal MRI in diagnosis of renal disorders especially in cases of oligohydramnios. This is supported by three of the studies concluding that fetal MRI was most useful in detection and assessment of severity in bilateral renal disease. Other studies that did not meet the criteria for inclusion in this paper have reported similar improvement in diagnostic accuracy for renal anomalies in both unilateral and bilateral pathologies [11, 22].

The overall scope of the review is limited predominantly by sample size as only twelve studies met the eligibility criteria and total number of patients included was 361. Whilst there is a reasonable amount of evidence concerning renal and genito-urinary problems (seven studies with 228 patients) some of the other conditions were only represented by a single study each. Furthermore, congenital lung malformations were only represented by two studies which seems discordant with clinical data as thoracic anomalies account for 5–18% of all congenital anomalies [23]. This may be due to the plethora of studies assessing various aspects of fetal MRI in cases of congenital diaphragmatic hernia but none looking at diagnostic accuracy. Studies that did not fit the criteria for inclusion suggest a range of diagnostic accuracy, however fetal MRI has been shown to be superior to ultrasound in most of these [24, 25]. Others have shown how lung lesions change over time making prediction of the histological type difficult [26], leading to many centres providing a description of the lesion at a certain point in time and not a diagnosis. The time period covered by these studies has seen significant evolution of both the quality of MRI scans and the ability of radiologists to interpret them. These improvements may limit study quality, however ultrasound image quality has also improved over this time.

These results have significant implications for future research to consolidate the evidence concerning improved diagnostic accuracy of fetal MRI. Improved diagnostic accuracy enables antenatal counselling to be tailored to each individual patient and will provide support for both parents and clinicians when making difficult decisions regarding the pregnancy. The additional information provided by fetal MRI could also aid in planning of delivery and the management of the neonate after birth.

The evidence provided by future larger studies could have an important role in the development of consensus both within the UK and internationally on the role that MRI should play in the diagnosis of congenital anomalies of the fetal body. Development of standardised protocols of how feto-maternal medicine units use MRI to aid diagnosis, parental counselling and antenatal management decisions will ensure this process is evidence based.

## Conclusion

In conclusion, this systematic review summarises the current evidence on the diagnostic accuracy of fetal MRI in diagnosis of non-CNS congenital anomalies compared with antenatal ultrasound alone. It shows an improvement in correct diagnosis up to 25.8% when MRI is used in addition to ultrasound with an odds ratio of 0.86. Antenatal ultrasound remains the gold standard in diagnosis of congenital anomalies of the fetal body, however fetal MRI can be used as an adjunct to provide further diagnostic information which may impact management.

However, the review is limited by the sample size of the studies with only single studies conducted for certain anatomical areas. Further research is needed to supplement these findings.

### Electronic supplementary material

Below is the link to the electronic supplementary material.

Supplementary Material 1

Supplementary Material 2

Supplementary Material 3

## Data Availability

The datasets used and/or analysed during the current study are available from the corresponding author on reasonable request.

## Abbreviations

PRISMA: Preferred Reporting Items for Systematic Reviews and Meta-Analyses
PROSPERO: International Prospective Register of Systematic Reviews
USS: Ultrasound Scanning
MRI: Magnetic Resonance Imaging
CNS: Central Nervous System
BMI: Body Mass Index
