# Supplementary material for: The value of fetal magnetic resonance imaging in diagnosis of congenital anomalies of the fetal body: a systematic review and meta-analysis
Source: BMC Med Imaging. 2024 May 16;24:111. doi: 10.1186/s12880-024-01286-5 (PMC11097489; doi:10.1186/s12880-024-01286-5)
Supplement: Supplementary file 1 — Supplementary Material 1 [file 12880_2024_1286_MOESM1_ESM.docx]

**Appendix S1**

Search strategy

Medline (via Ovid 1966-present)

| **Search no.** | **Query** | **Results (n)** |
| --- | --- | --- |
| **17** | #16 AND congenital anomaly | 392 |
| **16** | #8 AND #15 | 698 |
| **15** | #11 OR #12 OR #13 OR #14 | 5,324,440 |
| **14** | abdomen OR abdominal OR gastrointestinal OR GI tract | 1,494,932 |
| **13** | thoracic OR Chest OR Lungs | 1,563,924 |
| **12** | renal OR kidney | 1,259,757 |
| **11** | body | 1,569,160 |
| **10** | #8 NOT #9 | 1,391 |
| **9** | cardiac OR heart OR Cardiovascular system | 2,739,995 |
| **8** | #6 NOT #7 | 1,700 |
| **7** | CNS OR central nervous system OR Brain OR Spine | 2,758,241 |
| **6** | #4 AND antenatal diagnosis | 2,989 |
| **5** | #4 AND prenatal diagnosis | 2,928 |
| **4** | #1 AND #2 AND #3 | 4,903 |
| **3** | ultrasound or ultrasonography | 1,910,881 |
| **2** | MRI OR Magnetic resonance imaging | 723,986 |
| **1** | fetal[Title/Abstract] OR fetus[Title/Abstract] | 307,483 |
