# Supplementary material for: The value of fetal magnetic resonance imaging in diagnosis of congenital anomalies of the fetal body: a systematic review and meta-analysis
Source: BMC Med Imaging. 2024 May 16;24:111. doi: 10.1186/s12880-024-01286-5 (PMC11097489; doi:10.1186/s12880-024-01286-5)
Supplement: Supplementary file 2 — Supplementary Material 2 [file 12880_2024_1286_MOESM2_ESM.docx]

**Appendix S2**

Data collection tool

Author & Year

Title

Journal of publication

Country of study

Target population

Study characteristics:

Participant selection (consecutive, random or unclear)

Retrospective or prospective

Total no. in study

No. excluded by study authors + why

Total no. included in study report

Total no. included in systematic review

Gestation at USS

Gestation at MRI

Outcome reference standard used

Results:

USS result (% correct compared with outcome)

MRI result (% correct compared with outcome)

Analysis:

USS & MRI agreed + correct

USS & MRI agreed but wrong

MRI changed diagnosis (USS wrong)

USS changed diagnosis (MRI wrong)

Additional info given by MRI

Management changed by MRI

Specific anomaly where MRI most useful
